# Impact on ICSI outcomes of adding 24 h of *in vitro* culture before testicular sperm freezing: a retrospective study

**DOI:** 10.1186/s12610-015-0022-3

**Published:** 2015-06-09

**Authors:** Laurent Desch, Céline Bruno, Charlène Herbemont, Frédéric Michel, Shaliha Bechoua, Sophie Girod, Paul Sagot, Patricia Fauque

**Affiliations:** Laboratoire de Biologie de la Reproduction, Hôpital de Dijon, Université de Bourgogne, 2 Bd Mal De Lattre De Tassigny, 21079 Dijon, France; Service de Chirurgie Urologique-Andrologie, Hôpital de Dijon, Université de Bourgogne, Dijon, France; Service de Gynécologie-Obstétrique, Hôpital de Dijon, Université de Bourgogne, Dijon, France

**Keywords:** Culture, Freezing, ICSI, Outcome, Testicular sperm, Culture, Congélation, ICSI, Issues, Spermatozoïde testiculaire

## Abstract

**Purpose:**

To compare sperm parameters and intracytoplasmic sperm injection (ICSI) outcomes for testicular spermatozoa frozen on the day of the biopsy (DO) with those frozen after 24 h of *in vitro* culture (D1).

**Methods:**

In this retrospective study, from 1999 to 2012, forty-nine azoospermic patients were included to compare sperm (motility and viability) and outcomes (fertilization (FR), implantation (IR), pregnancy (PR) and delivery rates (DR)).

**Results:**

The *in vitro* culture increased total motility (+2.8 %, *p* = 0.0161) but decreased viability (−8.3 %, *p* = 0.007). After 24 h of culture, the post-thaw changes in motility and viability were not significant. Twenty-six couples underwent ICSI: thirty–four ICSI were performed with spermatozoa cryopreserved at D0 and eighteen with spermatozoa frozen at D1. Cumulated IR and DR were lower for ICSI with D1 spermatozoa than with D0 spermatozoa (IR: 21.6 % with D0 vs. 9.8 % with D1, *p* = 0.102; DR: 27.5 % with D0 vs. 8.3 % with D1, *p* = 0.049).

**Conclusion:**

Despite improving motility, freezing spermatozoa 24 h after testicular biopsy had a potential negative effect on ICSI outcomes, notably on delivery rates. These results may be related to the detrimental impact of the additional culture on the nuclear integrity of sperm.

## Résumé

**Objectif:**

Comparer les paramètres spermatiques et les issues de fécondation *in vitro* avec micro-injection (ICSI) de spermatozoïdes testiculaires congelés le jour de la biopsie (D0) avec ceux congelés après 24 heures de culture *in vitro* (D1).

**Méthodes:**

Dans cette étude rétrospective, de 1999 à 2012, quarante-neuf patients présentant une azoospermie ont été inclus pour comparer les paramètres spermatiques (mobilité et vitalité) et les issues d’ICSI (taux de fécondation (FR), d’implantation (IR), de grossesse (PR), et d’accouchement (DR)).

**Résultats:**

La culture *in vitro* augmentait la mobilité (+2.8 %, *p* = 0.0161) mais diminuait la vitalité (-8.3 %, *p* = 0.007). Après cumul des 24 heures de culture et congélation, les différences observées n’étaient plus significatives. Vingt-six couples ont eu au moins une ICSI : 34 ont été réalisées avec des spermatozoïdes congelés à D0 et 18 ont été réalisées avec des spermatozoïdes congelés à D1. Les taux d’implantation et d’accouchement cumulés étaient plus faibles avec les spermatozoïdes congelés à D1 par rapport à ceux congelés à D0 (IR: 21.6 % avec D0 vs. 9.8 % avec D1, *p* = 0.102; DR: 27.5 % avec D0 vs. 8.3 % avec D1, *p* = 0.049).

**Conclusion:**

Malgré l’augmentation de la mobilité, la congélation de spermatozoïdes testiculaires 24 heures après la biopsie apparait avoir un impact négatif sur les issues d’ICSI, notamment sur les taux d’accouchement. Ces résultats pourraient être en lien avec les effets néfastes de l’association des deux procédés (l’incubation pendant 24H cumulée à la congélation-décongélation) sur l’intégrité nucléaire spermatique.

## Background

Testicular sperm extraction (TESE) and ICSI have become common procedures in assisted reproduction programs for the treatment of both obstructive and non-obstructive azoospermia. As similar results in terms of fertilization, pregnancy and delivery rates are observed with the use of both frozen and fresh testicular spermatozoa [1], most centers freeze testicular spermatozoa retrieved after TESE and perform asynchronous ICSI. Indeed, this strategy makes it possible to prevent ICSI cancellations if no spermatozoa were retrieved [2].

ICSI achieved with testicular spermatozoa is acutely dependent on spermatozoa viability. However, their motility is often poor, making the selection of viable spermatozoa difficult. Therefore, several complementary approaches to sperm selection have been developed in order to detect immotile but live sperm or to optimize sperm function *in vitro* [3–10]. Among these is the ability of live sperm to respond to a hypoosmotic test, in which live sperm show swelling of the tail in hypoosmotic media [5]. A variety of substances, such as pentoxifylline, are also commonly used to stimulate sperm motility [3, 6, 8–10]. Some authors also advise promoting sperm motility by increasing the culture time as an alternative to viability tests. Indeed, in 1995, Craft and colleagues reported in the Lancet a significant increase in the number of progressively motile sperm after 24–72 h of *in vitro* culture in *in vitro* fertilization culture medium [11]. A few years later, other studies praised this practice [12–22]. Indeed, it has been reported that extended *in vitro* culture improves the motility of testicular sperm [12–20] with a maximum motility rate between 48 and 72 h of *in vitro* culture [17, 19, 20]. Based on these results, numerous teams around the world, like the previous head of Dijon’s Laboratory in 1999, developed this practice in laboratories. However, to date, only two studies on fresh cultured testicular spermatozoa have reported data on delivery rates [23, 24]. Moreover, there are currently no data available concerning the performance of ICSI using testicular spermatozoa frozen after an additional *in vitro* culture time of 24 h. In addition, some results in the literature suggest that prolonged *in vitro* incubation may have negative effects on nuclear status, in particular a deterioration in the integrity of sperm nuclei, which may potentially lead to adverse ICSI outcomes [25, 26].

Therefore, the aim of the present retrospective study was to analyze the interest and impact of an additional incubation period of 24 h followed by cryopreservation of testicular spermatozoa. For these purposes, we analyzed the testicular sperm parameters and we investigated ICSI outcomes associated with using testicular spermatozoa cryopreserved on the same day of retrieval or following 24 h of culture.

## Methods

### Patients

In this retrospective study, only patients with successful testicular sperm extraction (TESE) and with two cryopreserved sperm samples (one on the day of the testicular sperm retrieval (D0) and one the following day, after 24 h of supplementary *in vitro* culture (D1)) between January 1999 and December 2012 at Dijon University Hospital (France) were included.

During this period, 178 patients with obstructive (OA) and non-obstructive azoospermia (NOA) underwent testicular sperm extraction (TESE) at our center. Testicular sperm samples were successfully retrieved from 106 patients (59.6 %). For forty-nine (46.2 %) of these 106 patients, testicular sperm samples were cryopreserved at D0 and D1. Thus, semen analyses were based on 49 patients, 7 had NOA (14.3 %) and 42 had OA (85.7 %).

ICSI was carried out in 26 of the patients to study their outcomes: spermatozoa frozen at D0 (SPZ D0) was used for 17 patients, spermatozoa frozen at D1 (SPZ D1) was used for 5 patients and both testicular spermatozoa frozen at D0 and D1 was used for 4 patients on different ICSI cycles (identified as “the subgroup of same couples”). Of the 26 patients, 5 had NOA (19.2 %) and 21 had OA (80.8 %). The choice to use spermatozoa cryopreserved at D0 or D1 was based on the results of post-thawing tests.

Institutional Review Board approval was obtained for the collection of data of couples who had undergone ICSI cycles.

### Testicular sperm extraction and cryopreservation

The biopsies were obtained during open surgery under general anesthesia. Two testicular specimens of approximately 5 mm long and 5 mm thick were obtained from two different locations on the same testis. The biopsy samples were then independently minced using sterile needles in a dish containing 3 ml of Ferticult Hepes (Fertipro, Belgium) and incubated at 37 °C under 5.5 % CO_2_ and a humidified atmosphere for 30 min in IVF medium (Fertipro, Belgium) [27]. Each supernatant was then transferred to a 15 ml conical Falcon tube and diluted with a sperm cryoprotectant medium (SpermFreeze, Fertipro, Belgium) according to the manufacturer’s protocol. This semen sample was cryopreserved in freezing straws (Cryo Bio Systems, France). After being maintained at room temperature for 10 min, the straws were slowly cooled in liquid nitrogen vapour as described by Karacan *et al*. [24], and stored in liquid nitrogen at −196 °C.

After the addition of IVF medium (3 ml) to the remaining minced tissue, the culture was pursued for an additional 24 h in the same conditions. The next day (D1), the second supernatant was frozen using the same protocol as for the D0 sample.

The thawing test was performed the same day for both testicular sperm suspensions cryopreserved at D0 and D1 as follows: the straws were taken out of the liquid nitrogen and kept at room temperature (RT) for 8 min before draining into a test tube. Ten microliters of fresh and frozen-thawed testicular sperm at D0 and D1 were analyzed for concentration, motility, and viability using eosin-nigrosin smears [28].

### ICSI outcomes

#### ICSI processes

The female partners of these 26 patients underwent ovarian stimulation. Cryopreserved testicular spermatozoa were thawed on the same day as oocyte retrieval only if the presence of mature oocytes was confirmed after oocyte denudation. After thawing, the testicular spermatozoa were washed in 1 ml of culture medium (Ferticult flushing, Fertipro, Belgium) with centrifugation at 600 g for 5 min at RT. After centrifugation, the supernatant was removed and the pellet was resuspended in about 50 μL of culture medium with Hepes (Ferticult Hepes, Fertipro, Belgium). Then assisted fertilization by ICSI was performed as previously described [29].

#### ICSI evaluation

Fertilization was assessed 17–19 h after ICSI by checking the number of pronuclei. The fertilization rate was defined as the ratio between the number of diploid zygotes and the number of mature oocytes. Embryo cleavage was evaluated after 44 +/−1 h of culture. The morphological appearance of embryos was monitored according to the number and the size of the blastomeres (regular or irregular cleavage) as well as the percentage of anucleate fragments [30]. Embryos seen fertilized at day 1 with regular 4- to 5-cell embryos at day 2 with less than 20 % fragmentation and without any multinuclear blastomeres were regarded as “TOP” grade. The percentage of TOP embryos was defined as the ratio between the number of TOP embryos and the total number of embryos. Depending on the age of the women, the number of previous cycles, and the number and quality of embryos available, 1 or 2 embryos were transferred at either day 2 or day 3 after oocyte retrieval. Progesterone was administered vaginally every day from the day of oocyte retrieval until the time of a negative pregnancy test or until 8 weeks of gestation. Embryo cryopreservation and frozen-thawed embryo transfer were performed as previously described [31].

Clinical pregnancy was determined by the presence of an intrauterine gestational sac with a fetal heartbeat on ultrasound examination 4–5 weeks after the embryo transfer. The implantation rate was the ratio between the number of gestational sacs and the number of transferred embryos. The delivery rate was the ratio between the number of deliveries and the number of embryo transfers. In the same way, the outcomes of frozen embryo cycles were also analyzed.

### Statistical analysis

Quantitative variables were described using means and standard deviations, qualitative variables using frequencies and proportions. Sperm parameters before and after freezing-thawing as well as before and after the additional *in vitro* culture of 24 h were compared by means of Wilcoxon’s paired test.

The results for fresh embryo transfers alone or cumulated with those of frozen embryo transfers between spermatozoa cryopreserved at D0 and D1 were compared using the Chi-square test (or Fisher’s exact test, if the expected values were < 5). Data were analyzed using SAS version 9.2 in the Clinical Research Unity of the University Hospital of Dijon. Differences were considered significant when *p* < 0.05.

## Results

### Semen analysis

The results for the effects of freezing and/or *in vitro* culture on sperm parameters are presented in Table 1. Overall, freezing decreased the motility of spermatozoa cryopreserved at D0 or D1 (–6.0 % and –6.3 %, respectively; *p* < 0.001) and also their viability (–25.2 % and –20.8 %, respectively; *p* < 0.001). The *in vitro* culture increased total motility (+2.8 %, *p* = 0.0161) but decreased viability (–8.3 %, *p* = 0.007). This increase in motility after 24 h of supplemental *in vitro* culture was no longer significant after thawing (+1.4 %, *p* = 0.0895). Identical effects were found for testicular spermatozoa from NOA and OA patients. The mean number of frozen straws was 14.6 +/–8.5 (range: 3 to 34 straws) and 8.2 +/–4.5 (range: 2 to 23 straws) at D0 and D1, respectively.

**Table 1:** Effects of *in vitro* culture and freezing on sperm parameters

Parameters	Average (%) ± SD^a^	*p* value	^b^ *n*
Effect of freezing (difference after thawing-before freezing)			
Motility D0^c^	−6.0 ± 13.7	0.0003	40
Average motility after thawing D0	1.9 ± 4.8		
Average motility before freezing D0	7.9 ± 14.7		
Motility D1^d^	−6.3 ± 9.9	0.0003	43
Average motility after thawing D1	3.4 ± 7.6		
Average motility before freezing D1	9.7 ± 12.7		
Viability D0	−25.2 ± 18.3	<0.0001	40
Average viability after thawing D0	54.4 ± 16.5		
Average viability before freezing D0	79.6 ± 11.9		
Viability D1	−20.8 ± 15.6	<0.0001	33
Average viability after thawing D1	51.0 ± 18.9		
Average viability before freezing D1	71.8 ± 13.7		
Effect of *in vitro* culture (difference after *in vitro* culture-before *in vitro* culture)			
Motility	+2.8 ± 12.0	0.0161	47
Average motility after *in vitro* culture D1 before freezing	10.7 ± 13.55		
Average motility before *in vitro* culture D0 before freezing	7.9 ± 14.6		
Viability	−8.3 ± 13.8	0.0070	35
Average viability after *in vitro* culture D1 before freezing	71.0 ± 13.7		
Average viability before *in vitro* culture D0 before freezing	79.3 ± 12.4		
Effect of freezing and *in vitro* culture (difference D1-D0 after thawing)			
Motility	+1.4 ± 5.4	0.0895	44
Average motility after thawing D0	2.0 ± 4.9		
Average motility after thawing D1	3.4 ± 7.6		
Viability	−2.0 ± 21.6	0.7201	34
Average viability after thawing D0	54.0 ± 16.8		
Average viability after thawing D1	52.0 ± 17.7		

*No* Number of

^a^SD: Standard Deviation

^b^n: number of available data

^c^D0: Day of the biopsy

^d^D1: 24H after biopsy

### ICSI outcomes

Among the 49 patients included to study their sperm parameters, twenty-six underwent ICSI. Thirty–four ICSI were performed with spermatozoa cryopreserved at D0. Eighteen ICSI were performed with spermatozoa frozen at D1. Finally, for 4 couples, 12 ICSI treatments were done with both spermatozoa cryopreserved at D0 (4 ICSI) and D1 (8 ICSI), on different cycles.

The ICSI results obtained with spermatozoa frozen at D1 were compared with those obtained with spermatozoa frozen at D0 (Table 2).

**Table 2:** Baseline characteristics and ICSI outcomes

	ICSI with D0 or D1 Spz *in all couples*			ICSI with D0 and D1 Spz *among the same couples*		
	D0	D1	*p* value	D0	D1	*p* value
No. cycles	34	18		4	8	
Female age (y)	31.1	32.4	0.931	33.0	35.4	0.555
Male age (y)	38.7	38.1	0.658	35.0	36.6	0.801
Etiology of infertility: Male factor only (%)	64.7	61.1	0.798	75.0	50.0	0.576
No. collected oocytes (mean No oocyte/woman)	326 (9.6)	195 (10.8)	0.325	41 (10.3)	105 (13.1)	0.549
No. injected oocytes	269	130		32	70	
No. fertilized oocytes (%)	159 (59.1)	74 (56.9)	0.678	26 (81.3)	37 (52.9)	**0.006**
No. embryos (%)	186 (69.1)	82 (63.1)	0.226	29 (90.6)	44 (62.9)	**0.004**
No. “TOP” embryos (%)	73 (39.2)	37 (45.1)	0.368	15 (51.7)	13 (29.5)	0.057
No. fresh transfered embryos	63	27		8	13	
Fresh implantation rate (%)	23.8	11.1	0.167	37.5	15.4	0.248
Fresh pregnancy rate (%)	32.4	11.1	0.092	50.0	12.5	0.157
Fresh delivery rate (%)	29.4	5.6	**0.045**	50.0	12.5	0.157
No. cycles with cryopreserved embryos (%)	17 (50.0)	6 (33.3)	0.250	1 (75.0)	4 (37.5)	0.221
No. cryopreserved embryos (%)	66 (35.5)	23 (28.0)	0.234	13 (44.8)	10 (22.7)	**0.047**
No. cryopreserved embryos thawed and transfered	35	23		1	10	
Cumulated implantation rate (%)	21.6	9.8	0.102	40	17.6	0.201
Cumulated pregnancy rate (%)	29.4	12.5	0.092	60	16.7	0.074
Cumulated delivery rate (%)	27.5	8.3	**0.049**	60	16.7	0.074

*No* Number of, *y* years

Significant results in bold

For the same couples, fertilization, embryo cleavage and frozen embryo rates were significantly lower when spermatozoa frozen at D1 were used than was the case with spermatozoa frozen at D0 (Table 2). In the same couples as well as in all couples, the implantation, pregnancy and delivery rates were lower when spermatozoa frozen at D1 were used than when spermatozoa frozen at D0 were used. Among all couples, the delivery rate was significantly higher with spermatozoa frozen at D0 than with spermatozoa frozen at D1 (29.4 % (D0) vs. 5.6 % (D1), *p* = 0.045). No difference was found according to the type of azoospermia (OA vs NOA–data not shown). The cumulated delivery rates taking into account fresh and frozen embryo transfers were significantly higher for spermatozoa frozen at D0 (27.5 % (D0) vs. 8.3 % (D1), *p* = 0.049).

## Discussion

To the best of our knowledge, no study has assessed the impact on ICSI outcomes of an additional 24 h of *in vitro* culture before testicular sperm freezing. This study revealed that the best outcomes were achieved when the testicular sperm was frozen on the day of the testicular biopsy. Despite improving motility, the additional 24 h of culture before freezing had a negative effect on fertilization and implantation.

Our results confirmed that the *in vitro* culture of testicular spermatozoa for 24 h significantly improved their motility [11–20]. However, the increase in motility was no longer significant after thawing. Emiliani and collaborators [15] observed similar results when the testicular spermatozoa were cultured after thawing. In addition, as previously reported, we found that the freezing step decreased testicular sperm viability [27]. Thus, even though the second freezing step performed after 24 h of *in vitro* culture could increase the number of straws available for further ICSI attempts, the accumulation of both sperm treatment processes may be unfavorable to sperm quality/nuclear integrity.

In the current study, even though the number of ICSI cycles was relatively small, the significant results proved that the additional 24 h of culture followed by the freezing step had adverse effects on ICSI outcomes. Indeed, as highlighted in ICSI in the same couples, the fertilization rate was lower with testicular spermatozoa frozen one day after the biopsy than was the case with spermatozoa frozen immediately after the biopsy. To date, there are no data available concerning the efficacy of ICSI carried out with cultured and then frozen testicular spermatozoa. However, some authors have reported ICSI outcomes after testicular sperm cryopreservation alone. Although fertilization and pregnancy rates were similar [32], implantation rates were lower [1] and spontaneous abortion rates were higher [33] in ICSI cycles with frozen testicular spermatozoa than with fresh spermatozoa. Moreover, even though several authors have reported that extended *in vitro* culture improved the motility of testicular spermatozoa, very few studies have analyzed implantation, pregnancy and delivery rates following this treatment process [18, 23, 24, 34] (Table 3). Only one reported a significant improvement in the fertilization rate with the use of spermatozoa incubated for 24 h [18]. However, in this study with unfrozen cultured spermatozoa, the clinical pregnancy rate was very low as compared to the biochemical pregnancy rate (35.3 % vs 53.9 %, respectively), suggesting a high number of miscarriages with spermatozoa processed in this way, which agrees with our findings. In the other study, even though the results were not significant, the delivery rates were lower in ICSI with cultured testicular spermatozoa than in ICSI without an incubation step [23]. In addition, the sperm used in ICSI pregnancies followed by miscarriage displayed high levels of fragmentation: OR = 2.68 IC95: 1.40–5.14; *p* = 0.003 [35]. It would thus be interesting to know if miscarriage could be explained by a high level of sperm DNA fragmentation induced by *in vitro* culture.

**Table 3:** ICSI outcomes with or without cultured testicular spermatozoa

References	Studied group	Group size (No. ICSI)	Results			
			FR (%)	CPR (%)	IR (%)	DR (%)
Levran *et al.* 2001 [23]	1. TSPZ, fresh, no culture	23	61.7	34.8	13.9	26.1
	VS					
	2. TSPZ, fresh, 24 h culture	24	58.9	29.2	7.5	21.7
Windt *et al.* 2002 [18]	1. TSPZ, fresh, no culture	76	61.9	19.7		
	VS					
	2. TSPZ, fresh, 24 h culture	17	**73.7**	35.3		
Wood *et al.* 2003 [34]	1. TSPZ, fresh, no culture	35	62.3	23.0		
	VS					
	2. TSPZ, fresh, 24-48 h culture	38	71.1	24.0		
Karacan *et al.* 2013 [24]	1. TSPZ, fresh, no culture	166	70.7	31.3	13.4	28.9
	VS					
	2. TSPZ, fresh, 24 h culture	42	68.7	30.9	16.5	28.5
Our study	1. TSPZ, no culture, post-thawed	34	59.1	29.4	21.6	29.4
	VS					
	2. TSPZ, 24H culture, post-thawed	18	56.9	12.5	9.8	**5.6**

Significant results in bold

*CPR* clinical pregnancy rate, *DR* delivery rate, *FR* fertilization rate, *R* implantation rate, *No* Number of, *TSPZ* testicular spermatozoa, *VS* versus

The deleterious effects of culture on outcomes and more particularly on fertilization may be related to the detrimental impact on sperm integrity of Reactive Oxygen Species (ROS) produced by the spermatozoa themselves [36], and ROS production is known to be increased by culture [37]. In addition, even though no major changes were observed for chromatin condensation and acrosome reaction [37], severe structural chromosomal abnormalities (deletions, translocations, breaks, gaps, acentric fragments) were reported in *in vitro* stored ejaculated spermatozoa [38]. DNA damage assessed by the acridine-orange test on over 232 chromosome spreads from sperm samples of 2 donors revealed an excess of breaks in DNA after 24 h of *in vitro* culture [38]. Furthermore, DNA fragmentation measured with the TUNEL assay in testicular sperm from men with obstructive azoospermia was higher following long incubation than following short or no incubation [26]. In this latter work, the authors also reported an increase in DNA fragmentation even after the freezing step alone. Moreover, Matsuura *et al*. also described the influence of incubation temperature on DNA fragmentation: the temperature of 37 °C significantly increased the DNA fragmentation index compared with room temperature [39]. The addition of both procedures probably has a cumulative negative effect on sperm DNA.

## Conclusions

In light of these significant findings, we recommend that reproductive clinics do not prolong *in vitro* culture before testicular sperm freezing. Indeed, by handling gametes and especially by prolonging and potentiating sperm exposure to non-physiological conditions, extended *in vitro* culture followed by freezing may induce iatrogenic damage, including an increase in sperm DNA fragmentation and embryo development failure.

Finally, while the implication of testicular spermatozoa in birth defects is controversial [40], we wonder if the use of *in vitro* cultured then frozen testicular spermatozoa could have a negative impact on the health of children thus conceived. These infants would need to be followed in the long-term to detect any resulting anomalies or other unwanted effects.

## Abbreviations

D0: Day of the biopsy
D1: After 24 h of in vitro culture
DNA: Deoxyribonucleic acid
DR: Delivery rate
FR: Fertilization rate
ICSI: Intracytoplasmic sperm injection
IR: Implantation rate
NOA: Non-obstructive azoospermia
OA: Obstructive azoospermia
PR: Pregnancy rate
RT: Room temperature
ROS: Reactive oxygen species
SPZ: Spermatozoa
TESE: Testicular sperm extraction
WHO: World health organization
